# The Chinese Association for the Study of Pain (CASP): Expert Consensus on the Cervicogenic Headache

**DOI:** 10.1155/2019/9617280

**Published:** 2019-04-01

**Authors:** Hong Xiao, Baogan Peng, Ke Ma, Dong Huang, Xianguo Liu, Yan Lu, Qing Liu, Lijuan Lu, Jingfeng Liu, Yimei Li, Tao Song, Wei Tao, Wen Shen, Xiaoqiu Yang, Lin Wang, Xiaomei Zhang, Zhigang Zhuang, Hui Liu, Yanqing Liu

**Affiliations:** ^1^Department of Algology, West China Hospital of Sichuan University, Chengdu, Sichuan, China; ^2^Department of Orthopedics, General Hospital of Armed Police Force, Beijing, China; ^3^Department of Algology, Xinhua Hospital Affiliated to Shanghai Jiao Tong University School of Medicine, Shanghai, China; ^4^Department of Algology, The Third Xiangya Hospital of Central South University, Changsha, Hunan, China; ^5^Pain Research Center of Sun Yat-Sen University, Guangzhou, Guang Dong, China; ^6^Department of Algology, Xijing Hospital, Fourth Military Medical University, Xi'an, Shanxi, China; ^7^Department of Algology, The Affiliated T.C.M Hospital of Southwest Medical University, Luzhou, Sichuan, China; ^8^Department of Algology, Nanjing Drum Tower Hospital, The Affiliated Hospital of Nanjing University Medical School, Nanjing, Jiangsu, China; ^9^Department of Algology, The 2nd Affiliated Hospital of Harbin Medical University, Harbin, Heilongjiang, China; ^10^Department of Algology, The First Affiliated Hospital of Xinjiang Medical University, Wulumuqi, Xinjiang, China; ^11^Department of Algology, The First Affiliated Hospital of China Medical University, Shenyang, Liaoling, China; ^12^Department of Neurosurgery, Shenzhen University General Hospital, Shenzhen, Guangdong, China; ^13^Department of Algology, The Affiliated Hospital of Xuzhou Medical University, Xuzhou, Jiangsu, China; ^14^Department of Algology, The First Affiliated Hospital of Chongqing Medical University, Chongqing, China; ^15^Department of Algology, The First Affiliated Hospital of Guizhou Medical University, Guiyang, Guizhou, China; ^16^Department of Algology, The First Affiliated Hospital of Kunming Medical University, Kunming, Yunnan, China; ^17^Department of Algology, The Second Affiliated Hospital of Zhengzhou University, Zhengzhou, Henan, China; ^18^Department of Algology, Beijing Tiantan Hospital, Beijing, China

## Abstract

Cervicogenic headache is a relatively common but unique form of headache, and in China, as well as in several other countries, both diagnosis and a clear evidence-based treatment plan remain controversial. Therefore, the Chinese Association for the Study of Pain organized a meeting of pain management experts and created an expert consensus on the diagnosis and treatment of cervicogenic headache in China. This article summarizes the conclusions of the consensus group regarding the epidemiology, etiology, clinical features, diagnosis, differential diagnosis, treatment, and rehabilitation of cervicogenic headache in China.

## 1. Introduction

Although cervicogenic headache (CEH) is a common clinical challenge [1–3], controversies still exist regarding its diagnosis and treatment. Therefore, the Chinese Association for the Study of Pain organized a meeting of pain management experts from China to reach consensus on issues surrounding its diagnosis, treatment, and rehabilitation. The group comprised neurologists, orthopedists, and headache specialists. This article reflects the resulting expert consensus on the diagnosis and treatment of CEH in China. Suggestions are also proposed for consideration by headache specialists from other countries.

## 2. Definition

In 2013, the International Headache Society proposed the most recent definition for CEH [2]. According to this, CEH is defined as any headache caused by a disorder of the cervical spine or its components, such as bone, disc, and/or soft tissue elements, usually, but not invariably, accompanied by neck pain.

## 3. Diagnostic Criteria

CEH is typically diagnosed based on a detailed history, physical examination, and comprehensive evaluation of the nervous system. Disappearance of headache following a diagnostic block test supports a cervical source of the pain. The diagnostic criteria recommended by the International Classification of Headache Disorders (ICHD) 3rd edition [3] are as follows:

- Any headache fulfilling criterion (c)
- Clinical, laboratory, and/or imaging evidence of a disorder or lesion within the cervical spine or soft tissues of the neck that can cause headache
- Evidence of causation demonstrated by at least two of the following findings:
  1. Headache has developed in temporal relation to the onset of cervical disorder or appearance of the lesion
  2. Headache has significantly improved or resolved along with improvement in or resolution of cervical disorder or lesion
  3. Cervical range of motion is reduced, and headache is significantly aggravated by provocative maneuvers
  4. Headache disappears after diagnostic block to the suspected cervical spine structure or its supply nerve
- Not better accounted for by another ICHD-3 diagnosis

## 4. Epidemiology

The prevalence of CEH varies in the general population depending on the diagnostic criteria used. However, it has been estimated that 1.0%–4.1% of the population experience CEH [4–6]. Data have revealed that it even ranged to 17.5% in patients with severe headache [7]. Better-controlled and larger-scale epidemiological studies are needed to clarify the true prevalence of CEH.

## 5. Etiology

### 5.1. Anatomy

Recent studies have indicated that head and face pain in CEH originate from disorders of the upper cervical nerves (C1–C3) [8]. Indeed, lesions in the atlantooccipital joint, atlantoaxial joint, C2-C3 zygapophyseal joint, and intervertebral disk can all cause occipital pain [9–11]. In contrast, there is no evidence that regions innervated by the lower cervical nerves directly induce headache. Thus, the spinal nerves and branches from C1–C3 are thought to be the main anatomical bases of CEH.

The C1 spinal nerve (suboccipital nerve) innervates the atlantooccipital joint. Pathological change in or damage to this joint may cause pain to be referred in the occipital region. The C2 spinal nerve innervates the atlantoaxial and C2-C3 zygapophyseal joints and is adjacent to the lateral portion of the articular capsule of the C1-C2 zygapophyseal joint (atlantoaxial joint). Pathological changes or injures in these joints or the surrounding tissues can lead to referred pain in the head. The third occipital nerve (C3 medial branch) approaches and dominates the C2-C3 zygapophyseal joint, from where pain can radiate to the occipital, frontal, temporal, and periorbital regions (third occipital neuropathic headache) of the head.

Although the pain resulting from each pathology is not always consistent among patients, the distribution range of pain is typically similar. Neck pain originating near the cranium can radiate to the head, including the frontal and periorbital regions. Conversely, neck pain originating from the pars caudalis of the upper three cervical spines always causes pain in the occipital region. Moreover, a study showed that impairment of the C2-C3 zygapophyseal joint causes CEH in 70% of patients [12], of whom 27% can be diagnosed with third occipital neuropathic headache. Deterioration of the C3-C4 zygapophyseal joints, upper cervical intervertebral discs, and lower cervical zygapophyseal joints are less common causes of CEH.

### 5.2. Pathophysiology

**Figure 1:**
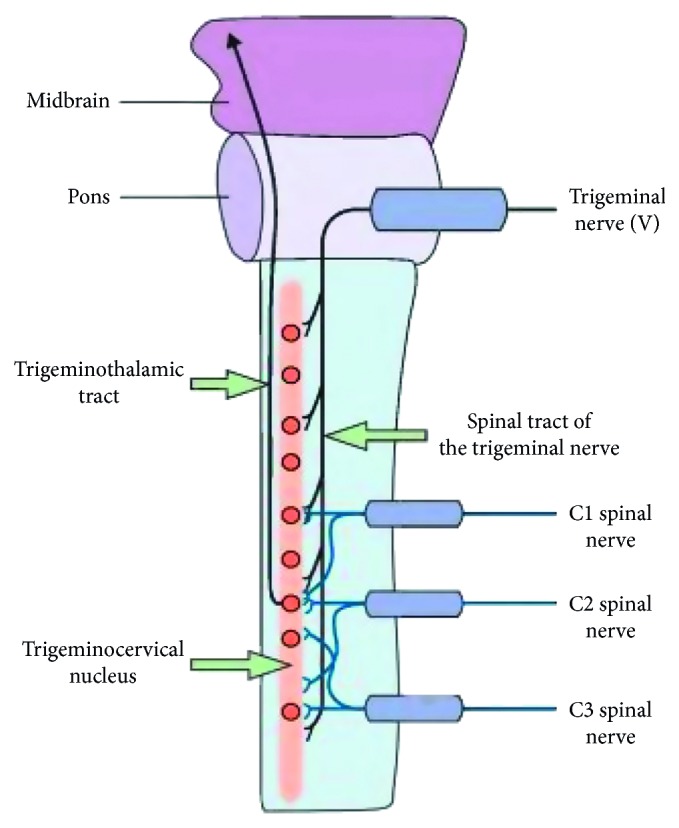
Mechanism of pain referral from the cervical spine to the head.

The pathophysiology of CEH is poorly understood, but it is thought to be a referred pain originating from pathological changes in the upper cervical zygapophyseal joints. As shown in Figure 1 [1], anatomical convergence of pain fibers from the trigeminal nerve (including the ophthalmic division) and the upper three cervical nerves forms the basis for pain to be referred from the upper cervical region to the head, including radiation to the frontal and periorbital regions [13, 14]. The trigeminocervical nucleus receives not only the C1–C3 afferents but also the first branch of the trigeminal sensory afferents, indicating that it receives second-order neuron afferents from the trigeminal and upper three cervical spinal nerves. Therefore, pathological changes in the cervical zygapophyseal joints can generate pain in the areas innervated by either the trigeminal nerve (e.g., frontal and periorbital regions) or the upper three cervical spinal nerves (e.g., occipital and ear regions). Convergence between the trigeminal sensory descending tracts and the upper cervical nerve roots can also cause referred pain in the neck, face, and head.

## 6. Clinical Features

### 6.1. Symptoms

1. CEH is a chronic unilateral headache; it is also a side-locked headache [15].
2. Pain is first noted in the neck or occipital region before it radiates to the ipsilateral frontotemporal and orbital regions. Temporal regions are the most commonly affected.
3. CEH is usually a deep, blunt, distending, and tense pain without pulsation. The frontotemporal region is the most painful, and the headache is aggravated by neck movements, fatigue, or an unhealthy neck position and relieved by rest.
4. Headache may intermittently occur and last for hours or days, but during the late stages, it can cause persistent pain.
5. Stiffness and a restricted range of motion in the neck may occur and be accompanied by ipsilateral shoulder or arm pain.
6. Most patients also have concomitant symptoms of nausea, tinnitus, dizziness, phonophobia, photophobia, blurred vision, or disordered sleep.

### 6.2. Physical Examination

Patients with CEH are more likely to have myofascial trigger points on the transverse processes of the second cervical vertebra that can spread to the head and splenius capitis, trapezius, sternocleidomastoid, and suboccipital muscles [16]. Tenderness is observed in the occiput, paravertebral muscles, mastoid process, unilateral or bilateral outlets of the greater occipital nerve, and transverse process of the third cervical vertebra [17]. Patients often present with a limited range of motion of the cervical spine. There is no tenderness of the head and face.

### 6.3. Investigations

Magnetic resonance imaging (MRI) may show cervical disc degeneration, herniation, or bulging, mostly in the discs of C2–C5. Radiographs may show degenerative changes in the atlantoaxial, zygapophyseal, and uncovertebral joints [18]. However, radiography, MRI, and computed tomography (CT) are typically of limited value in the diagnosis of CEH.

## 7. Differential Diagnosis

### 7.1. Primary Headache

Primary headaches can be identified by their typical clinical manifestations [19].

1. Tension-type headache has the following characteristics: (1) bilateral; (2) pressing or tightening (nonpulsating) pain; (3) paroxysmal pain; (4) not aggravated by routine physical activity; (6) no nausea or vomiting; (7) can be aggravated by compression of the frontal, temporal, masseter, pterygoid, sternocleidomastoid, splenius, and trapezius muscles.
2. Migraine has the following characteristics: (1) unilateral, (2) pulsating pain, (3) headache lasting 4–72 h, (4) aggravation by routine physical activity (e.g., walking or climbing stairs), (5) accompanied by nausea/vomiting/photophobia/phonophobia, (6) ipsilateral premonitory visual symptoms (e.g., flashing, darkness, lines, or blindness), or sensory symptoms (e.g., numbness) lasting >5 min, (7) ergotamine or triptans can be effective, and (8) alleviated by pregnancy.

### 7.2. Secondary Headaches

1. Posttraumatic headache can be diagnosed based on its clinical manifestation and MRI and CT findings.
2. Vascular headache, including poststroke, hemangioma, and hypertensive headaches, can be diagnosed based on its clinical manifestation and MRI (including angiography) and CT (including CT or digital subtraction angiography) findings.
3. Headache caused by high or low intracranial pressure. High intracranial pressure is usually caused by tumor or inflammation, which can result in a persistent, nonpulsatile, and severe headache with papilledema and emesis. Low intracranial pressure is usually caused by lumbar puncture, which can be relieved after lying down and aggravated by sitting in an upright posture.
4. Referred headache from skull structures, such as eyes, ears, sinuses, and teeth (e.g., glaucoma, sinusitis, and periodontitis). Specialist examination can aid in the diagnosis, such as intraocular pressure determination, sinus CT, and dental examination.
5. Somatoform disorders should also be considered. Physical and auxiliary examinations are normal in these patients. Repeated and changing physical symptoms are presented, and pain areas are not fixed. The patient may also have anxiety and depression.

## 8. Treatment

### 8.1. Pharmacotherapy

Despite a lack of convincing clinical studies on effective medications for CEH, pharmacotherapy remains among the best available treatments [20, 21]. Several types of medications are commonly used [22, 23]:

1. Nonsteroidal anti-inflammatory drugs (NSAIDs) can be effective, including nonselective COX and selective COX-2 inhibitors.
2. Muscle relaxants, particularly tizanidine, baclofen, and eperisone hydrochloride, that have central mechanisms of action can also provide analgesic effects in both the acute phase and for prevention. Tizanidine can be combined with NSAIDs because of its gastroprotective effect and good safety.
3. Antiepileptic drugs and antidepressants can be used in patients with neuropathic pain. Common drugs for this purpose include gabapentin, pregabalin, amitriptyline, venlafaxine, and duloxetine.

### 8.2. Injection to the Atlantoaxial and C2-C3 Zygapophyseal Joints and Nerve Block

Individualized injection therapy can be selected according to the pain's location and characteristics. These procedures are associated with risks of structural damage from needle placement but can also result in the injection of local anesthetic into the vertebral artery, high-level epidural anesthesia, total spinal cord anesthesia, or injury of the spinal cord and nerve roots. Nonparticulate, water-soluble steroid hormones are recommended to prevent the embolism of steroid hormone particles.

#### 8.2.1. Joint Injection

*(1) Atlantoaxial Joint Injection*. This can benefit patients with suboccipital or occipital pain aggravated by cervical rotation [24] or pain due to inflammatory stimuli [25]. Intraarticular injection can use a lateral or posterior approach and should be slow, with the volume not exceeding 1 ml. One study has shown that injection to the atlantoaxial joint was effective in 81.2% of cases. After 3 months' follow-up, 20% of patients had pain relief exceeding 50% [26].

*(2) C2-C3 Zygapophyseal Joint Injection*. This injection can be considered for patients with upper neck pain spreading to the occipital region or pain that increases when the neck is rotated or back is stretched. During the procedure, the head can be rotated for a better field of vision. Again, the injection should be slow and should not exceed a total volume of 1 ml. However, the therapeutic efficacy of C2-C3 intra-articular injection remains controversial [27–29].

#### 8.2.2. Nerve Block

*(1) Cervical Spinal Nerve Root Block*. Selective nerve root injection could be used in patients with cervical spondylotic radiculopathy although the depth of the needle should be monitored to prevent complications. The effectiveness of this method is 70%, with 50% still reporting relief after 12 months [30].

*(2) Third Occipital Nerve Block*. This block can be used to diagnose CEH and predict the efficacy of radiofrequency treatment.

*(3) Occipital Nerve Block*. This block can be used to diagnose and treat occipital pain [21]. Clinically, occipital nerve injection can be repeatedly and intermittently used for symptomatic treatment. Dose for diagnostic injection should be limited to 2 ml; however, when it reaches 3–5 ml, the greater and small occipital nerves are completely blocked.

### 8.3. Minimally Invasive Interventional Management

Radiofrequency therapy, including radiofrequency thermocoagulation and pulse radiofrequency, is minimally invasive interventional techniques. Nerve properties can be distinguished by monitoring the stimulation frequency and impedance. Interventional radiofrequency therapy can be considered if a diagnostic nerve block is effective [31].

Radiofrequency intervention is recommended for patients with intractable cervical headache from the C2-C3 zygapophyseal joint for which conservative treatment has failed and complete relief has been obtained from a diagnostic nerve block [13]. The efficacy for CEH of other origins needs to be confirmed. Pulse radiofrequency is a type of neuromodulation therapy that has shown satisfactory short-term efficacy, albeit with recurrence during long-term follow-up that requires repeated treatments [32]. The reason for poor performance of C2 ganglion pulse radiofrequency treatment in some patients may be occipital pain from the C1 and C3 spinal nerve branches through the superior cervical plexus. Percutaneous laser disc decompression is another effective minimally invasive procedure for patients with cervical disc herniation, protrusion, or disc degeneration who have neck and shoulder pain with nerve root symptoms. Also, plasma radiofrequency can be considered when other methods fail to achieve satisfactory results by reducing the volume of the nucleus pulposus, potentially improving symptoms.

Finally, ozone has strong anti-inflammatory and analgesic effects that can benefit patients in whom glucocorticoid use is contraindicated. Its efficacy can be further improved through combination with a nerve block. Clinical effectiveness of percutaneous radiofrequency ablation combined with ozone is superior to that of percutaneous radiofrequency ablation alone in the treatment of cervical disc herniation, providing better medium- and long-term outcomes. Percutaneous laser disc decompression, ozone therapy, and other techniques have shown some clinical efficacy.

### 8.4. Surgical Procedures

Surgery is not usually recommended for CEH, except when CEH is refractory and does not respond to noninvasive treatment, and there is convincing evidence that CEH is caused by pathological changes amenable to surgical treatment. Surgery may be beneficial for three specific causes of CEH as follows: (1) C2 spinal nerve compression by vascular/ligamentous structures, (2) osteoarthritis of the lateral atlantoaxial joint, and (3) upper cervical intervertebral disc pathology [33].

### 8.5. Physical Therapy

Physiotherapy is almost universally available and risk-free and has enormous benefits [34–36], such as reducing the frequency of CEH and improving headaches in the long-term. In view of the noninvasive nature of physiotherapy, it is recommended that it be the first choice for patients with CEH. Options include manipulation therapy (e.g., pulling, relaxation, and chiropractic), specific training therapy (e.g., static and dynamic stretching and training), and low-load endurance muscle exercise focusing on the neck and shoulder joints or the upper limbs (moderately strong evidence) [37–39]. In the initial stages, the muscles are gently stretched and artificial neck traction is performed to make physical therapy easier for the patient. Next, strength training and aerobic exercise are gradually introduced according to the patient's tolerance. High-velocity manipulation is not recommended because of the risk of interregional arterial dissection and stroke.
